# Hypotension and hypovolemia during hemodialysis: is the usual suspect innocent?

**DOI:** 10.1186/s13054-016-1307-4

**Published:** 2016-06-09

**Authors:** David Berger, Jukka Takala

**Affiliations:** Department of Intensive Care Medicine, Inselspital, Bern University Hospital, University of Bern, CH-3010 Bern, Switzerland

## Abstract

Hypotension during intermittent hemodialysis is common, and has been attributed to acute volume shifts, shifts in osmolarity, electrolyte imbalance, temperature changes, altered vasoregulation, and sheer hypovolemia. Although hypovolemia may intuitively seem a likely cause for hypotension in intensive care patients, its role in the pathogenesis of intradialytic hypotension may be overestimated.

## Background

In the current issue, Bitker et al. [1] report the prevalence and risk factors of hypotension during intermittent hemodialysis in ICU patients. Hypotension during intermittent hemodialysis occurs in around 50–60 % of critically ill patients [1, 2] and in 25–50 % of chronic renal failure patients [3]. Its pathophysiology is multifactorial and incompletely understood. In intensive care patients, intradialytic hypotension may cause problems in volume management and metabolic control.

Therapeutic interventions to correct hypotension, if necessary, should be oriented to the pathophysiology rather than to symptoms alone. In chronic hemodialysis patients, risk factors of hypotension include shifts in extracellular volume, osmolarity, and electrolytes, dialysis-induced temperature changes, and altered vasoregulation [3]. Their relevance in ICU patients undergoing hemodialysis is less clear. Even small volume shifts may cause hypotension in ICU patients with limited cardiovascular reserves. Hence, acute volume shifts or sheer hypovolemia and decreased cardiac output may intuitively seem to be likely causes of intradialytic hypotension in the ICU. This assumption is contradicted by Bitker et al. Although hypotension was common (in 57 % of 107 sessions), only every fifth hypotensive episode was associated with preload dependence, defined as increased stroke volume following passive leg raising (PLR) [1]. The hypotensive episodes were frequent despite proactive control of dialysate temperature and sodium concentration and limited blood flow, known risk factors of intradialysis hypotension [1, 2]. Since hypotension occurred early and after minor ultrafiltration, altered vasomotor tone was considered the likely explanation. This is consistent with observations during chronic hemodialysis [4]. Accordingly, reduced ultrafiltration would not be the most rational intervention, although frequent in clinical practice.

Preload dependence or a change in cardiac output in response to preload alteration is normal, whereas preload independence is pathologic, indicating that the heart is operating at the flat part of its function curve. Hence, preload dependence does not indicate a need for more volume [5]. Preload is the muscle tension before contraction, best assessed as end-diastolic pressure [6]. The apparent preload independence could be due to three mechanisms: lack of true increase in preload by PLR, ongoing vasodilation, or cardiac function limitation. Let us consider these mechanisms:

1. PLR is assumed to shift a volume of up to 300 ml in the absence of overt hypovolemia [7]. Volume overload in acute kidney injury is very frequent [8]. Lack of volume shift in response to PLR therefore seems rather unlikely.
2. PLR during ongoing vasodilatation may fail to increase preload. As in the study of Bitker et al., hypotension during chronic hemodialysis occurs early and independently of ultrafiltrate volume, and is related to decreased vasomotor tone [3, 4].
3. Finally, true cardiac function limitation with fluid overload [8] could have been present: the heart works on the flat part of its function curve, and the circulation is not limited by venous return. In addition to fluid overload, acute kidney injury may induce myocardial dysfunction via various cytokines [9]. Myocardial stunning during hemodialysis with transient wall motion abnormalities was recently recognized in chronic kidney disease [10].

The median change in cardiac output observed by Bitker and coworkers from prehemodialysis values to hypotension was almost zero [1], but the individual responses were very heterogeneous (increases/decreases >12.5 % in 20/48 instances). Hence, the pathophysiology of hypotension appears more complex. Notably, preload dependence *before* hemodialysis was only present in five instances. Volume overload [8] and decreased cardiac function [9, 10] must have contributed to this. Preload assessment before and during hemodialysis and individual responses would have allowed clarification of the hemodynamic mechanisms involved.

## Conclusion

We congratulate Bitker et al. on this study because it challenges common clinical practice. We fully support their conclusion that intradialytic hypotension “should not necessarily lead to reduction of fluid removal by hemodialysis but should prompt hemodynamic evaluation of cardiovascular status to reliably classify patients regarding their preload dependence status” [1]. Hypervolemia and impaired cardiac function were almost certainly highly prevalent [8–10]. Because hemodialysis can severely impair tissue perfusion without overt clinical signs [11] and fluid dynamics during ultrafiltration might be considerably different between ICU patients and chronic hemodialysis [12, 13], individual hemodynamic assessment could be more comprehensive than PLR alone. The assessment should include the clinical context, the hemodynamic status pre dialysis, and an independent judgment if a preload maneuver shows the expected effects.

## Abbreviation

PLR: passive leg raising

## References

[1] Bitker L, Bayle F, Yonis H, Gobert F, Leray V, Taponnier R (2016). Prevalence and risk factors of hypotension associated with preload-dependence during intermittent hemodialysis in critically ill patients. Crit Care.

[2] Schortgen F, Soubrier N, Delclaux C, Thuong M, Girou E, Brun-Buisson C. Hemodynamic tolerance of intermittent hemodialysis in critically ill patients: usefulness of practice guidelines. Am J Respir Crit Care Med. 2000;162(1):197-202. 10.1164/ajrccm.162.1.9907098 10903241

[3] Reilly RF (2014). Attending rounds: a patient with intradialytic hypotension. Clin J Am Soc Nephrol.

[4] Power A, Charitaki E, Davenport A. Changes in vascular tone occur early during hemodialysis treatments independently of volume reduction. Artif Organs. 2015. [Epub ahead of print]. 10.1111/aor.12610 26496182

[5] Takala J. Volume responsive, but does the patient need volume? Intensive Care Med*.* 2016. [Epub ahead of print]. 10.1007/s00134-015-4172-8 26825955

[6] Magder S (2015). Understanding central venous pressure: not a preload index?. Curr Opin Crit Care.

[7] Aneman A, Sondergaard S. Understanding the passive leg raising test. Intensive Care Med. 2016. [Epub ahead of print]. 10.1007/s00134-016-4228-4 26846515

[8] Wang N, Jiang L, Zhu B, Wen Y, Xi XM (2015). Fluid balance and mortality in critically ill patients with acute kidney injury: a multicenter prospective epidemiological study. Crit Care..

[9] Shiao CC, Wu PC, Huang TM, Lai TS, Yang WS, Wu CH (2015). Long-term remote organ consequences following acute kidney injury. Crit Care.

[10] Burton JO, Jefferies HJ, Selby NM, McIntyre CW (2009). Hemodialysis-induced cardiac injury: determinants and associated outcomes. Clin J Am Soc Nephrol.

[11] Jakob SM, Ruokonen E, Vuolteenaho O, Lampainen E, Takala J (2001). Splanchnic perfusion during hemodialysis: evidence for marginal tissue perfusion. Crit Care Med.

[12] Ismael S, Savalle M, Trivin C, Gillaizeau F, D’Auzac C, Faisy C (2014). The consequences of sudden fluid shifts on body composition in critically ill patients. Crit Care.

[13] Schortgen F. Hypotension during intermittent hemodialysis: new insights into an old problem. Intensive Care Med. 2003;29(10):1645-9. 10.1007/s00134-003-1945-2 13680118

